# Femoral Neck Fracture in a Pediatric Patient with Primary Hyperparathyroidism

**DOI:** 10.1155/2023/5550451

**Published:** 2023-10-09

**Authors:** Mark W. Schmitt, Maxwell J. Modrak, Soumar J. Bouza, Brian G. Smith, Murillo A. Adrados

**Affiliations:** ^1^Department of Orthopaedic Surgery, Institute for Orthopaedics & Neurosciences, Carilion Clinic, 2331 Franklin Road Southwest, Roanoke, Virginia 24014, USA; ^2^Department of Orthopaedics and Rehabilitation, Yale University School of Medicine, 20 York Street, New Haven, Connecticut 06510, USA; ^3^Department of Pathology, Yale University School of Medicine, 20 York Street, New Haven, Connecticut 06510, USA; ^4^Department of Orthopaedic Surgery, Texas Children's Hospital, Main Campus, 6701 Fannin Street, Houston, Texas 77030, USA

## Abstract

**Case:**

A previously healthy 11-year-old girl underwent expedited surgical fixation of a femoral neck fracture sustained while jump-roping. After further work up, she was diagnosed with primary hyperparathyroidism. Parathyroidectomy of a hypertrophic adenoma proved curative. Now, five months post left hip surgery, the patient is pain-free and walks without a limp.

**Conclusion:**

We describe the first published case of primary hyperparathyroidism presenting as a pathologic hip fracture in a child. Although presentation with a fracture is exceedingly rare, bone pain is a frequent complaint of pediatric hyperparathyroidism. Orthopedic surgeons may find themselves the front-line caregivers for the condition.

## 1. Introduction

Primary hyperparathyroidism (PHPT) is seldom reported in children, with the majority of cases occurring in adolescence due to the sporadic development of a parathyroid adenoma resulting in the failure to inhibit the release of parathyroid hormone (PTH) in the presence of excess calcium [1]. PHPT is an infrequent cause of hypercalcemia in pediatric patients with an incidence of 2-5 per 100,000 patients [2]. PHPT in the adult population is commonly an asymptomatic disorder discovered incidentally through routine lab testing [3, 4], but in the juvenile population, PHPT is typically symptomatic and associated with other morbidity, such as kidney stones, abdominal pain, and skeletal fragility [5].

Osteitis fibrosa cystica (OFC) is an osseous manifestation resulting from PHPT due to prolonged exposure to high levels of serum PTH [6]. Bone involvement is reported in about 33-58% of children with any type of hyperparathyroidism [1, 7, 8], but due to improved early detection of PHPT, OFC is becoming increasingly rare with a reported prevalence of less than 2% [9]. OFC commonly presents in long bones with the radiographic finding known as a brown tumor [10]. These lesions are caused by bone demineralization due to excessive osteoclast activation, microfractures, and hemorrhages [10], resulting in subsequent weakening of the cortical bone matrix and increasing the risk for pathologic fractures [3, 10, 11]. In children, most pathologic fractures are secondary to benign tumors, while only a small portion are due to metabolic disorders or malignant tumors [12].

Pediatric femoral neck fractures represent a rare but emergent pathology that, if not treated quickly, can result in lasting disability [13–16]. Accounting for less than 1% of all fractures in children, femoral neck fractures are usually caused by high-energy trauma, but pathologic fractures and stress fractures have been identified in low-energy cases [16, 17]. The most common complication of femoral neck fractures is avascular necrosis, though this complication is highly dependent on both Delbert classification and patient age [13, 14, 16, 18]. The Delbert classification is described as one of four types depending on the location of the fracture. Type I fractures are transphyseal and have the highest rate of avascular necrosis [13, 14, 16, 17]. Type II is transcervical, type III is basicervical, and type IV is intertrochanteric [13, 14, 16–18]. Other feared complications include nonunion, premature physeal closure, and coxa vara/valga [17].

## 2. Case Presentation

**Figure 1:**
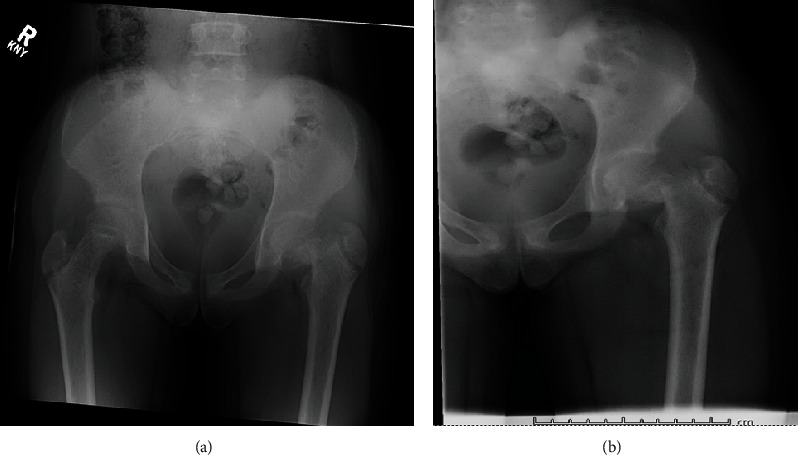
Initial X-rays demonstrating a Delbert type III left-sided femoral neck fracture.

The patient and her family were informed that data regarding their case would be submitted for publication. Since the patient was a minor, consent was given by the patient's mother. Our patient is a previously healthy 11-year-old girl with three days of limping and left groin pain. She fell after her left leg “gave” while jumping rope at school. She presented to an outside hospital where radiographs were consistent with a left femoral neck fracture. (Figure 1) The patient was then immediately transferred to a pediatric tertiary care center for definitive orthopaedic treatment.

**Figure 2:**
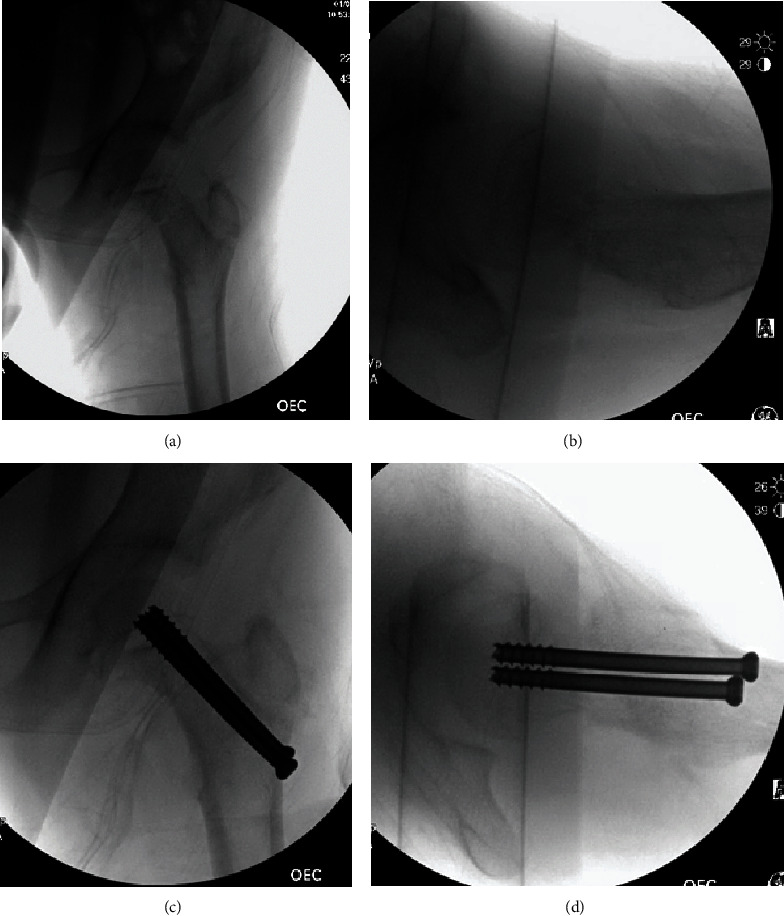
Intraoperative fluoroscopy demonstrating closed reduction utilizing a traction table and placement of two 6.5 mm screws for fixation.

Upon admission to the pediatric tertiary care center, bloodwork was drawn, and her operative fixation was expedited. She underwent close reduction-internal fixation an hour after arrival. The surgery was performed with the patient on a traction table, and anatomical reduction was achieved with gentle traction and internal rotation of the injured limb. Two 6.5 mm screws were used to compress and fix the fracture following anatomic reduction. (Figure 2) An anterior hip capsulotomy was also performed intraoperatively to relieve the expected high intracapsular pressure. Due to the osteopenic quality of the bone on radiographs, in addition to widened physis and the low energy mechanism of injury, a metabolic bone disease was suspected [19, 20]. To that end, two bone biopsies from the fracture site were obtained prior to fixation: one from drill hole reaming and a second from a Jamshidi bone needle. These samples later proved to be consistent with normal cancellous bone, both lamellar and woven. The wound was irrigated and closed in standard layered fashion. The patient tolerated the procedure well and awoke from anesthesia without issues. She was then admitted to the pediatric surgical floor, where pediatrics, pediatric surgery, and pediatric endocrine services were consulted for assistance in treatment and further work-up.

**Figure 3:**
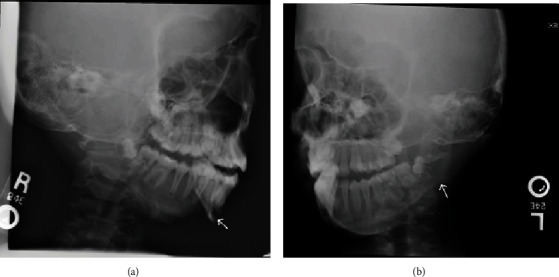
Mandibular X-rays on hospital day 2 displayed a lytic lesion with cortical breakthrough adjacent to the mandibular symphysis and a small area of erosion at the mandibular angle.

Given that metabolic bone disease was suspected, blood work was drawn to aid in diagnosing its origin. The patient's admission serum laboratory values were remarkable for severe serum hypercalcemia of 14.1 mg/dL (normal 8.8-10.2 mg/dL), ionized calcium of 7.48 mg/dL (normal 4.65-5.28 mg/dL), and hypophosphatemia of 2.7 mg/dL (normal 3.5-5.3 mg/dL). She was also found to have a low serum concentration of vitamin D 25-hydroxy at 8 ng/dL (normal between 20 and 50 ng/mL), with a high activated vitamin D 1,25-hydroxy of 102 (normal 25-66 ng/mL). Her alkaline phosphatase level was 3946 U/L (normal 50-480 U/L). Primary hyperparathyroidism was then definitively diagnosed with an elevated serum parathyroid hormone of 1874.0 (normal 10.0-69.0 pg/mL). Because of the high rate of tumor associations (multiple endocrine neoplasia syndromes) and other downstream consequences of hyperparathyroidism, several other studies were obtained. Bilateral renal ultrasounds showed no renal stones or adrenal gland masses (pheochromocytoma). Radiographs of the mandible were also taken and showed perimandibular lytic lesions (Figure 3). Plasma catecholamine and metanephrine levels that were drawn for workup of pheochromocytoma were within normal limits.

**Figure 4:**
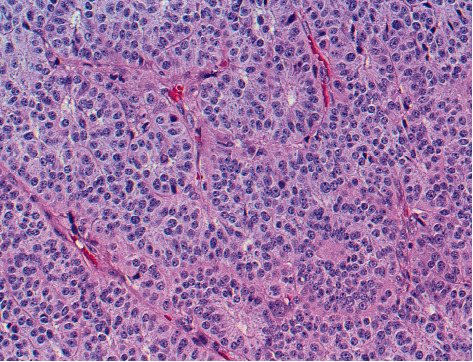
Hematoxylin and eosin stain at 100x magnification of the resected parathyroid gland demonstrating a well-circumscribed with mass with eosinophili cells that exhibit large atypical nuclei with prominent nucleoli.

**Figure 5:**
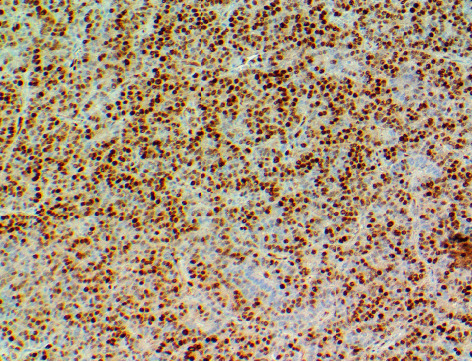
Cyclin D1 staining of the resected parathyroid gland shows diffusely positive staining for cyclin D1.

Postoperatively, the patient remained tachycardic, with a heart rate between 120 and 130, with hypertension to 120 mmHg systolic over 70 mmHg diastolic. Her cardiovascular symptoms were believed to be related to hypercalcemia. The patient's hypercalcemia was temporarily treated with hydration, diuretics, and calcitonin. A neck ultrasound revealed a 3 cm mass in the right lobe of the thyroid, consistent with a parathyroid mass. The patient underwent parathyroidectomy of the enlarged parathyroid gland on hospitalization day 6. Preliminary pathology revealed this was an atypical parafibromin-deficient parathyroid tumor (Figure 4). Further pathologic testing of the parathyroid mass revealed increased cyclin D1 expression (Figure 5), and genetic testing revealed that the patient had a CDC73/HRPT2 mutation. Following parathyroidectomy, the patient was again sent to the pediatric intensive care unit for close electrolyte management. As expected, she developed rebound hypocalcemia due to hungry bone syndrome, requiring the placement of a central line for calcium supplementation. Due to persistent metabolic derangement requiring close electrolyte management, the patient remained in the intensive care unit until hospital day 16. On hospital day, 20 a DEXA scan was done for an assessment of bone quality. AP spine (L1-L4) resulted a BMD of 0.576 g/cm^2^ and a *Z*-score of -1.6. Right femoral neck BMD of 0.488 g/cm^2^ and *Z*-score of -2.7. Right total hip BMD of 0.494 and *Z*-score of -2.9. 1/3 right forearm BMD of 0.398 g/cm^2^*Z*-score of -3.3. These results indicated that our patient had severely osteoporotic bone and would need therapy to improve bone quality. She was subsequently discharged to home on oral calcium supplementation.

**Figure 6:**
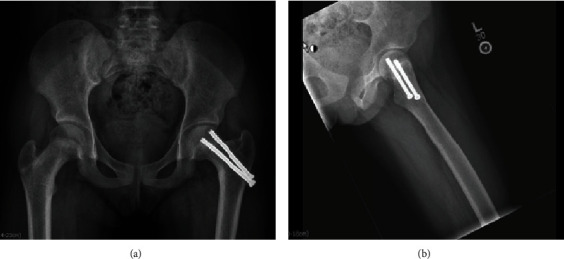
X-rays taken at 5 months postop demonstrate healing of the femoral neck fracture in some varus with some shortening of the femoral neck.

The patient's recovery was uncomplicated, and by the first outpatient appointment at 14 days after discharge, she reported no more pain. The patient was kept on crutches for 6 weeks and was not seen again until 5 months after surgery at which point she was full weight-bearing, without a limp, and had resumed all activities, including school gym (Figure 6). The patient and her family were non-English speaking immigrants, and despite a concerted effort by the medical team for additional follow-up, they did not return to the clinic after the 5th month from injury, when the patient was symptom-free.

## 3. Discussion

To our knowledge, this is the first reported case of primary hyperparathyroidism presenting as a pediatric pathologic hip fracture. This is an unusual presentation for a rare disorder but highlights teachable points. The fracture is atypical for a pediatric patient and is much more consistent with that or an acute femoral neck fracture in an elderly patient from a low-energy fall, and thus should raise suspicion of additional pathology. Had radiographs had been taken at the onset of pain osteopenia and a widened physis [21, 22] would have been found led to an orthopaedic referral or further diagnostic testing, potentially sparing her from this injury.

PHPT is typically diagnosed before it is severe enough to result in a pathological fracture. Children will often present with symptoms of hypercalcemia such as polyuria, fatigue, poor appetite, weight loss, abdominal pain, nausea, and vomiting [2]. Osseous pathology usually manifests later in the disease as bone pain, tenderness, gait disturbances, and decreasing height due to vertebral compression [1]. Our patient only had pain for three days prior to her fracture, and her symptoms were not severe enough to stop her from participating in recess at school. Her lack of symptoms aside from a limp is quite unusual for pediatric PHPT.

Treatment for PHPT involves resection or removal of the source of excessive parathyroid hormone (PTH), as well as treating pathology stemming from the hypercalcemic state. Parathyroidectomy is the treatment of choice for children with PHPT, but since PHPT is more severe in the pediatric population, there is an increased risk for hungry bone syndrome such as what occurred in this patient [23]. All pediatric patient diagnosed with PHPT should also be evaluated for multiple endocrine neoplasia (MEN) type 1 as part of their treatment, prompting a renal ultrasound and serum metanephrines [5].

In conclusion, skeletal manifestations, let alone a pathologic fracture, are less common with PHPT than in the past. Bone lesions caused by PHPT serve as a potential locus for pathologic fractures, which may present with a pattern not typically seen in pediatric patient populations. Fractures such as those of the femoral neck in pediatric patients represent an orthopaedic emergency, but the underlying pathology leading to these fractures requires significant attention.

## Data Availability

The case study data used to support the findings of this study have not been made available because the information presented in the case study is personal health information protected by an institutional review board.
